# 

**Published:** 1895-01-19

**Authors:** 


### CONTENTS.

**The Problem of the Poor** - - - - - - - - - - 269
**Education and Disease** - - - - - - - - - - 270
**On Modern Progress in Ophthalmic Medicine and Surgery.**—By R. Brudenell Carter, F.R.C.S. - - - 271
**Adventures of a Tenth Century Medical Student** - - 272
**Annotations.**—The Oyster Scare—Cycling and Heart Disease - 273
**Medical Progress and Hospital Clinics:**
Progress in Medicine.—Diseases of Children - - - - 275
Progress in Surgery.—Renal Surgery.—Rectal Surgery - - 276
New Appliances and Things Medical - - - - - 280
**Notes and News** - - - - - - - - - - 274
**The Medical Congress at Calcutta** - - - - 280
**The Institutional Workshop:**
The Financial Strength of Our Voluntary Charities - 281
Post Office and the Work of the Blind - - - 282
The Radcliffe Infirmary - - - - - - - 282
Hospital Festivals and Meetings - - - - - - 284
Practical Points - - - - - - - - - 284
**The Book World of Medicine and Science** - - - - 285
**The Student of Medicine** - - - - - - - 285

### EXTRA SUPPLEMENT.—THE NURSING MIRROR.

**News from the Nursing World.**—The Temperance Hospital.—Little Invalids.—Annual Taxation.—Metropolitan Asylums Board Nurses.—A Nurses' Home Wanted.—Trained Nurses at Dundee.—Primitive Dressings and Diets.—American Women as Professors.—Without Names.—Registration of Midwives.—Who Does the Work?—"The Hospital" Convalescent Fund.—Infirmary Nursing.—Short Items - - cxv

**Elementary Anatomy and Surgery for Nurses.** By W. McAdam Eccles, M.B., B.S., F.R.C.S. - - - - - cxvii

**The Madhouse of Fiction** - - - - - - - - cxviii

**The Glasgow Western Infirmary** - - - - - - cxix

**Wants and Workers** - - - - - - - - - cxix

**Nursing in Rio de Janeiro** - - - - - - - cxx

**Helps Homewards** - - - - - - - - - cxx

**The Lambeth Infirmary** - - - - - - - - cxx

**Our American Letter** - - - - - - - - cxxi

**The Muses' Looking Glass** - - - - - - - cxxii

**Entertainments** - - - - - - - - - - cxxiii

**Where to Go** - - - - - - - - - - cxxii

**Notes and Queries** - - - - - - - - - cxxii

**Nursing in Dublin—Presentations** - - - - - cxxiii

**Everybody's Opinion** - - - - - - - - cxxiv

